# Sleep quality and stress as influences on college students’ physical activity participation: a cross-sectional study

**DOI:** 10.3389/fpubh.2025.1640974

**Published:** 2025-10-13

**Authors:** Hao Wang, Shiqi Zhao, Tianle Fang, Lishun Xiao, Dehui Yin, Zhiming Sun

**Affiliations:** ^1^School of Public Health, Xuzhou Medical University, Xuzhou, China; ^2^Jiangsu Engineering Research Center of Biological Data Mining and Healthcare Transformation, Xuzhou Medical University, Xuzhou, Jiangsu, China

**Keywords:** college students, sleep quality, physical activity, stress, health behaviors

## Abstract

**Objective:**

This study examines the relationship between sleep, stress, and exercise among college students, as these factors are significantly associated with their well-being, academic performance, and quality of life. Understanding their interconnections is crucial for promoting healthy behaviors.

**Methods:**

Conducted from March to April 2025 at Xuzhou Medical University, the study involved distributing 840 electronic questionnaires to undergraduate, graduate, and doctoral students, yielding 796 valid responses (94.76% response rate). We used the Physical Activity Rating Scale-3 (PARS-3), Perceived Stress Scale (PSS-14), and Athens Insomnia Scale (AIS) to assess physical activity, stress, and sleep quality, respectively. Statistical analyses included multifactorial ANOVA, Spearman’s correlation, mediation analysis, and multiple linear regression using SPSS 27.0.

**Results:**

Male students had higher participation in medium- and large-scale sports than females. Second-year students showed lower participation rates. Obese or overweight students had higher participation in these sports. Both sleep quality and stress were significantly associated with exercise levels. There was an inverse relationship between exercise levels and stress, and between exercise levels and sleep quality. Stress was involved in the association between sleep quality and exercise level, confirmed by mediation analysis. Multiple linear regression further supported the significant association of stress and sleep quality with exercise levels.

**Conclusion:**

Sleep quality and stress levels are key factors associated with exercise participation among college students. Tailored health education and interventions are essential to improve students’ well-being. Future longitudinal studies are needed to establish causality and explore additional influencing factors.

## 1 Introduction

Sleep, stress, and exercise are essential components of a healthy lifestyle for college students (1). Sleep, as a fundamental physiological process, significantly influences both the physical and mental well-being of this demographic (2). Sleep deprivation can lead to a myriad of adverse effects on cognitive functioning, emotional stability, and physical health (3). For instance, it can negatively impact academic performance and contribute to a decline in overall health (4, 5). In contemporary society, college students face pressures from various sources, including academic demands, social interactions, and career planning (6), which render them particularly vulnerable to sleep disorders (7). Empirical studies indicate that a substantial percentage of college students experience sleep-related issues, which profoundly affect their academic performance and overall quality of life (8).

The impact of stress on college students is a significant concern that warrants attention (9). Prolonged exposure to high levels of stress not only disrupts the academic and daily lives of college students (10), but it can also precipitate various mental health issues, including anxiety and depression (11). These psychological challenges, in turn, may adversely affect sleep quality and participation in physical activities, thereby establishing a detrimental cycle.

Engagement in exercise, recognized as a beneficial health behavior, not only contributes to the enhancement of physical fitness but also plays a significant role in alleviating stress and improving sleep quality (12). Regular physical activity increases the body’s metabolic rate (13), regulates the endocrine system (14), and facilitates the release of neurotransmitters, including endorphins (15). Furthermore, consistent exercise can assist college students in mitigating stress levels and enhancing mental resilience, which subsequently leads to improved sleep quality (16). Nevertheless, the current state of physical activity among college students is unsatisfactory and is influenced by various factors, including demanding academic schedules and a lack of awareness regarding the importance of exercise (11).

In conclusion, it is imperative to examine the relationship between exercise as a healthy behavior and the factors influencing sleep and stress. This study seeks to analyze the intrinsic connections among these variables through a thorough and detailed investigation. The objective is to establish a robust foundation for promoting healthy behaviors among college students and for developing scientifically sound and effective health interventions, ultimately aiding college students in achieving optimal physical and mental development.

## 2 Materials and methods

### 2.1 Data sources

From March to April 2025, a comprehensive cluster sampling was conducted across the undergraduate, graduate, and doctoral programs at Xuzhou Medical University. A total of 840 electronic questionnaires were distributed in March 2025, and upon collection, invalid responses—characterized by incomplete basic information and missing content—were excluded from the analysis. Consequently, 796 valid questionnaires were retained, resulting in an effective response rate of 94.76%.

### 2.2 Research methodology

#### 2.2.1 Physical Activity Rating Scale-3 (PARS-3)

The PARS-3 scale was utilized to assess the level of physical activity among college students, as indicated in the existing literature. This scale quantifies exercise by measuring three key dimensions: intensity, duration, and frequency of participation in physical activities.

The quantity of exercise can be expressed as the product of intensity, duration, and frequency. Intensity and duration of activities are categorized into five levels, with frequency recorded as 1 to 5 points and duration recorded as 0 to 4 points. A total score of 19 or less indicates low levels of exercise, a score ranging from 20 to 41 indicates moderate levels of exercise, and a total score of 42 or higher indicates high levels of exercise. Previous studies have demonstrated that the test–retest reliability of each item on the PARS-3 questionnaire ranges from 0.838 to 0.942, indicating that the instrument possesses good reliability (17, 18).

#### 2.2.2 Perceived Stress Scale (PSS-14)

According to the relevant literature, this study employs the PSS-14 to assess the stress levels of college students. This scale comprehensively evaluates and quantifies various dimensions, including the frequency and intensity of stress experienced by individuals over the past month, as well as the degree of its impact on their lives. The PSS-14 comprises 14 items, with responses categorized into five levels, ranging from 0 to 4 points. A score of 0 signifies 1 indicates “almost never (a few times a year or less), 2 denotes “sometimes (once a month or less), 3 represents “often (a few times a month), and 4 corresponds to “always (every day). The scores for the 14 items are summed, yielding a total score that ranges from 0 to 56 points. Specifically, a total score of 0 to 19 points indicates a low stress level, a score of 20 to 39 points reflects a medium stress level, and a total score of 40 points or higher signifies a high stress level. In prior studies, the Cronbach’s alpha for this scale has been reported to be greater than 0.70 (19).

#### 2.2.3 Athens Insomnia Scale (AIS)

This study employs the AIS to assess the sleep status of college students, as supported by relevant literature. The AIS provides a quantitative evaluation across multiple dimensions closely associated with sleep, including sleep onset time, sleep maintenance, sleep quality, and daytime functioning. The scale comprises eight items, each categorized into four levels, ranging from 0 to 3 points, based on the individual’s circumstances. Specifically, a score of 0 indicates the absence of sleep problems, 1 signifies mild sleep issues, 2 denotes moderate sleep problems, and 3 reflects severe sleep problems. The scores from the eight items are summed, yielding a total score that ranges from 0 to 24 points. A total score of ≤3 points indicates no sleep disorder; scores between 4 and 6 suggest suspected insomnia; and a total score exceeding 6 points indicates severe insomnia. Previous research has reported an average reliability coefficient of 0.86 for the AIS-8 (20).

The objective of this study is to enhance the demographic information and other survey items pertaining to students. The independent variables include gender, grade level, height, and weight. Obesity was assessed using the body mass index (BMI) according to established classification standards for BMI for screening overweight and obesity in Chinese school-age children and adolescents categorizes BMI as follows: a BMI of less than 18.5 is classified as underweight, a BMI ranging from 18.5 to 23.9 is considered normal, a BMI between 24.0 and 26.9 is classified as overweight, and a BMI of 27.0 or greater is categorized as obese (21).

### 2.3 Statistical analysis

The SPSS 27.0 statistical software was employed to conduct a comprehensive analysis of the differences in exercise intensity, sleep quality, and anxiety levels across various demographic indicators. Qualitative data were presented as percentages (%) or constituent ratios (%). The chi-square (*χ*^2^) test was utilized for univariate analysis. Multivariate analysis of variance was applied to assess the homogeneity of variances for multi-factor analysis. Additionally, Spearman’s correlation analysis, multiple linear regression analysis, and mediating effect analysis were conducted to examine the factors influencing exercise levels, with a significance level set at *α* = 0.05. Data management and analysis were performed in April 2025.

## 3 Results

### 3.1 Common methodological biases

To mitigate prevalent methodological biases, this study employed the collection of anonymous responses and the reverse scoring of specific items. An exploratory factor analysis of the 25 items across the three scales, utilizing the Harman one-way test, revealed that a total of five factors exhibited an eigenvalue greater than 1. The variance accounted for by the first factor was 28.756%, which falls short of the critical threshold of 40%. Consequently, it can be concluded that there was no significant common method bias present in this study.

### 3.2 General characteristics of participants

A total of 796 valid questionnaires were collected for this survey, comprising 312 responses from male students and 484 from female students. The basic demographic information collected included grade level, field of study, and other relevant factors (see Table 1).

**Table 1:** Basic characteristics of participants.

Variables		Number	Rate (%)	Group		Number	Rate (%)
Gender	Male	312	39.2	Major	Clinical medicine	269	33.8
	Female	484	60.8		Preventive Medicine	289	36.3
					Pharmacy	6	0.8
					Nursing	89	11.2
Grade	First year	215	27.0		Medical Laboratory Technology	3	0.4
	Second year	114	14.3		Medical Imaging	67	8.4
	Third year	285	35.8		Others	71	8.9
	Fourth year	109	13.7	Body type	Obesity	53	6.7
	Fifth year	28	3.5		Fat	113	14.2
	Postgraduate	42	5.3		Thin	101	12.7
	Doctor	3	0.4		Normal	529	66.4

### 3.3 Physical activity of survey respondents

The findings of the study indicated that among the 796 university students surveyed, the proportion of male students engaging in medium- and large-sized sports (55.4%) was significantly higher than that of female students participating in the same categories (42.2%) (*χ*^2^ = 13.460, *p* < 0.01). Additionally, the participation rate in medium- and large-sized sports among second-year students (43.0%) was lower compared to first-year students (56.7%) and students in their third year or beyond (44.1%). Furthermore, the percentage of students classified as obese or overweight who participated in medium- and large-sized sports (53.0%) exceeded that of students with normal weight (48.8%) and those classified as underweight (30.7%). Detailed results are presented in Table 2.

**Table 2:** Physical activity among university students.

Variables		Small exercise (%)	Medium exercise (%)	Large exercise (%)	*χ* ^2^
Gender	Male	139 (44.6)	65 (20.8)	108 (34.6)	42.242
	Female	280 (57.9)	132 (27.3)	72 (14.9)	
Grade	First year	93 (43.3)	63 (29.3)	59 (27.4)	10.530
	Second year	65 (57.0)	25 (21.9)	24 (21.1)	
	Third year or more	261 (55.9)	109 (23.3)	97 (20.8)	
Body type	Thin	70 (69.3)	17 (16.8)	14 (13.9)	15.404
	Normal	271 (51.2)	139 (26.3)	119 (22.5)	
	Fat and obesity	78 (47.0)	41 (24.7)	47 (28.3)	
Sleep quality	No obstacle	58 (35.6)	39 (23.9)	66 (40.5)	45.628
	Minor obstacle	62 (48.1)	39 (30.2)	28 (21.7)	
	Insomnia	299 (59.3)	119 (23.6)	86 (17.1)	
Stress situation	Low stress level	100 (38.2)	77 (29.4)	85 (32.4)	38.912
	Medium stress level	314 (59.4)	120 (22.7)	95 (18.0)	
	High stress level	5 (100.0)	0	0	

### 3.4 Multifactor analysis of variance (ANOVA)

The total scores for the PARS-3, PSS-14, and AIS scales were computed and subsequently entered into a multifactorial variance model for analysis. The results of the analysis indicated that the overall model was significant, with a Type III sum of squares of 320,969.769, degrees of freedom of 366, a mean square of 876.967, an *F*-value of 1.473, and a significance level of less than 0.001. This suggests that the independent variables of sleep quality and stress status significantly influenced the dependent variable of exercise level. Specifically, the analysis of the relationship between sleep quality and exercise level revealed a Type III sum of squares of 41,613.520, degrees of freedom of 23, a mean square of 1,809.283, an *F*-value of 3.038, and a significance level of less than 0.001, indicating a significant impact of sleep quality on exercise level. In contrast, the examination of the relationship between stress status and exercise level yielded a Type III sum of squares of 40,152.413, degrees of freedom of 38, a mean square of 1,056.642, an *F*-value of 1.774, and a significance level of 0.004, demonstrating that stress status also significantly affected exercise level. Detailed results are presented in Table 3.

**Table 3:** Between-subjects effects test.

Source	Type III sum of squares	df	Mean square	*F*	Significance
Corrected Model	320969.769^a^	366	876.967	1.473	<0.001
Intercept	164078.369	1	164078.369	275.506	<0.001
Stress situation	41613.520	23	1809.283	3.038	<0.001
Sleep quality	40152.413	38	1056.642	1.774	0.004
Error	255492.325	429	595.553		
Total	1238437.000	796			
Corrected total	576462.094	795			

a *R*-squared = 0.557 (Adjusted *R*-squared = 0.179).

### 3.5 Spearman correlation analysis

The total scores from the PARS-3, PSS-14, and AIS scales were analyzed using Spearman’s rank correlation coefficient, which revealed a correlation coefficient of −0.222** between the level of exercise and sleep quality. Spearman’s correlation coefficient ranges from −1 to 1, with a negative value indicating an inverse relationship between the two variables. Specifically, a higher score on the exercise level scale corresponds to an increased amount of exercise, while a higher score on the Ascens Sleep Scale reflects more pronounced sleep disturbances. This negative correlation suggests that as the level of exercise increases, the quality of sleep tends to decrease; conversely, lower exercise levels are associated with poorer sleep quality. The absolute value of the correlation coefficient, 0.222, indicates a weak strength of correlation between exercise levels and sleep quality.

The correlation coefficient between exercise level and stress is −0.250**, indicating a negative correlation. This suggests that as the level of exercise increases, the level of stress is likely to decrease, and conversely, as the level of exercise decreases, the level of stress is likely to increase. Furthermore, the absolute value of 0.250 indicates a weak correlation between the two variables.

The correlation coefficient between sleep quality and stress status was found to be 0.372**, with a significance level (two-tailed) of less than 0.001. This indicates a significant positive correlation; specifically, higher scores on the Ascens Scale, which measures sleep disorders, are associated with greater levels of stress as indicated by the Stress Scale. Conversely, lower scores on the Ascens Scale correspond to lower levels of stress. It is important to note that the relationship between sleep disorders and stress may be influenced by a variety of complex factors, and should not be interpreted as a straightforward causal relationship.

All correlations are statistically significant (two-tailed) at a *p*-value of less than 0.001, and the accompanying remarks indicate that these correlations also achieve significance at the 0.01 level (two-tailed). This finding suggests that the correlations are so statistically robust that it is highly unlikely they are attributable to random variation. Consequently, there is substantial evidence to support the existence of a genuine association among the three variables: exercise level, sleep quality, and stressful situations. Detailed results are presented in Table 4.

**Table 4:** Spearman correlation analysis.

Variables	Exercise level	Sleep quality	Stress situation
Exercise level	1.000		
Sleep quality	−0.222**	1.000	
Significance (Two–tailed)	<0.001		
Stress situation	−0.250**	0.372**	1.000
Significance (Two–tailed)	<0.001	<0.001	

**Correlation is significant at the 0.01 level (Two-tailed).

### 3.6 Mediation analysis

**Figure 1:**
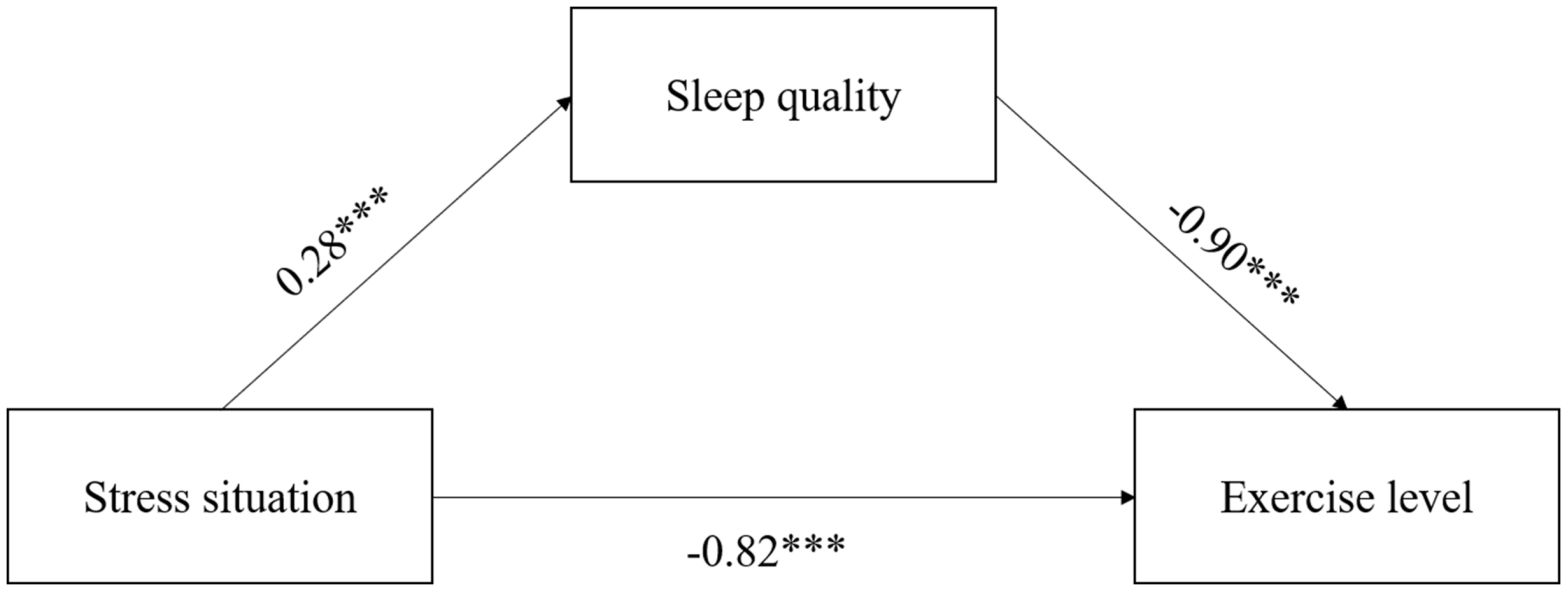
Path coefficient plots for stressful situations, sleep quality, and exercise level. *p<0.05, **p < 0.01, ***p < 0.001.

To investigate the intrinsic mechanism underlying the significant inverse relationship between sleep quality and exercise level, stress was introduced as a mediating variable within the structural equation model of this study. Utilizing Model 4 from the SPSS macro program Process, the mediating effect of stress on the relationship between sleep quality and exercise level was tested and analyzed using the Bootstrap method as outlined by Hayes. The coefficients representing the relationships among the three variables-stress situation, sleep quality, and exercise level—are illustrated in Figure 1.

According to Table 5, the upper and lower limits of the bootstrap 95% confidence intervals for the mediating effect of sleep quality on exercise level and stress situation do not include zero. This finding suggests that the stress situation not only exerts a direct effect on exercise level but also mediates the effect on exercise level through the variable of sleep quality. The direct effect, quantified as −0.82, was found to be significantly different from the mediating effect of sleep quality on exercise level, which was measured at −0.26. Collectively, the direct effect and the mediating effect accounted for 76.64 and 24.30% of the total effect, respectively, which was calculated to be −1.07.

**Table 5:** Decomposition table of total effect, direct effect and mediating effect.

Benefit type	Effect value	Se	LLCI	ULCI	Effect size
Total effect	−1.07	0.13	−1.33	−0.81	
Direct effect	−0.82	0.14	−1.09	−0.54	76.64%
Mediating effect	−0.26	0.06	−0.37	−0.15	24.30%

The path coefficient between stressful situations and sleep quality was found to be 0.28∗∗∗. This indicates that a higher score on the PSS-14 correlates with increased stress levels, which in turn is associated with poorer sleep quality as measured by the AIS. Specifically, as stress levels rise, sleep quality deteriorates. Furthermore, the path coefficient between sleep quality and exercise level was determined to be −0.90∗∗∗. This suggests that a higher AIS score, indicative of poorer sleep quality, corresponds to a lower score on the PARS-3, reflecting reduced levels of physical exercise. In other words, as sleep quality declines, exercise levels also decrease. Additionally, the path coefficient between stressful situations and exercise level was calculated to be −0.82∗∗∗. This finding indicates that higher scores on the PSS-14, which signify greater stress, are associated with lower scores on the PARS-3, suggesting a reduction in exercise levels. Thus, it can be concluded that increased stress is linked to decreased physical activity.

### 3.7 Multiple linear regression analysis (MLRA)

The total scores for the three scales—PARS-3, PSS-14, and AIS—were calculated individually. Initially, exercise level was designated as the dependent variable. Subsequently, age and BMI data were incorporated into a multifactorial ANOVA model, referred to as Model 1. The analysis revealed that the correlation coefficient (R) for Model 1, which included age and BMI, was 0.132. In contrast, the correlation coefficient (R) for Model 2, which additionally included stress and sleep quality, was 0.337. This indicates that the linear relationship between the independent variables and the dependent variable was stronger in Model 2.

The *R*-squared value for Model 1 is 0.017, indicating that the independent variable accounts for 1.7% of the variance in the dependent variable. In contrast, Model 2 has an *R*-squared value of 0.113, which explains 11.3% of the variance. The *R*-squared value was adjusted to account for the number of independent variables, thereby providing a more accurate assessment of the model’s goodness-of-fit. The adjusted *R*-squared value for Model 2 is 0.109, which is greater than the adjusted *R*-squared value of 0.015 for Model 1. This suggests that Model 2 offers a relatively better fit for the data.

The change in *R*-squared, the change in *F*-statistic, the degrees of freedom, and the significance of the change in *F*-statistic were all less than 0.001, indicating that the inclusion of the independent variables significantly enhanced the model. The detailed results are presented in Table 6.

**Table 6:** Multiple linear regression analysis.

Model	*R*	*R*-squared	Adjusted *R*-squared	Adjusted *R*-squared	Change in R-squared	*F*-change	df1	df2	Significance *F*-change
Model 1 (Age, BMI)	0.132	0.017	0.015	26.727	0.017	7.011	2	793	<0.001
Model 2 (Age, BMI, sleep quality, Stress situation)	0.337	0.113	0.109	25.420	0.096	42.808	2	791	<0.001

## 4 Discussion

### 4.1 Main findings and interpretation

Sleep, stress, and exercise are significantly associated with the physical and mental well-being of college students, which in turn affects their capacity for effective study and active living (22). In contemporary society, college students encounter various pressures, including academic, social, and career-related challenges, which are correlated with prevalent sleep issues (23). It is essential to investigate the factors that are associated with their sleep quality and physical activity, as well as to implement preventive measures. The findings of this study indicate notable differences in sports participation among college students based on gender, academic year, and body composition. The one-way analysis of variance (chi-square test) revealed that a higher proportion of male students engaged in medium- and large-scale sports compared to their female counterparts. This disparity may be linked to societal expectations regarding sports participation for different genders, as well as the inherent enthusiasm of male students for competitive sports. Furthermore, the data indicated that second-year students participated in medium and large sports at a lower rate than first-year and third-year students. This trend may be associated with the increased academic demands and course load encountered in the second year, which may limit time available for physical activity. Conversely, a higher proportion of obese and overweight students participating in medium and large sports may be linked to a stronger motivation to lose weight and enhance their health status.

This study initially employed multifactorial ANOVA to demonstrate that both sleep quality and stress levels are significantly associated with exercise participation. Subsequent Spearman correlation analysis revealed a clear relationship among sleep quality, exercise, and stress. Specifically, the findings indicate that higher levels of exercise are associated with lower stress levels and improved sleep quality; conversely, lower stress levels are associated with enhanced sleep quality. The significant negative correlation between exercise levels and stress serves as compelling evidence for the beneficial link of exercise to stress alleviation. The correlation coefficient between exercise levels and sleep quality was found to be −0.222**, indicating a statistically significant but weak correlation. Nonetheless, exercise and immunomodulation are interconnected and mutually associated with one another through various physiological mechanisms. Exercise is linked to immune regulation by affecting leukocytes, erythrocytes, cytokines, and other factors (24). Furthermore, physical activity has been recognized as an effective complementary therapeutic strategy associated with mood regulation and psychological improvement, thereby contributing to stress reduction (25–27). Lowered stress levels are linked to a conducive environment for restorative sleep. When an individual’s stress is diminished, both psychological and physiological tensions are alleviated, which is associated with facilitating entry into a relaxed sleep state, and this in turn is correlated with improved sleep quality (28). Additionally, physical activity can be linked to sleep promotion by modifying body composition (29), and appropriate levels of physical activity have been shown to be associated with enhanced cognitive function in adolescents (30). This paper also employed mediation analyses to further substantiate the findings that increased stress is correlated with poorer sleep quality, elevated stress is associated with reduced exercise levels, and diminished sleep quality is linked to lower levels of exercise.

This paper employs regression analysis to demonstrate that the association of age and Body Mass Index (BMI) with exercise levels are not statistically significant. This lack of significance may be linked to the limited age range among college students, as the sample consists predominantly of participants within a narrow age bracket. Consequently, it is challenging to discern variations in exercise levels that may be associated with differences in age. Additionally, the participants exhibit minimal variation in physiological functions, further complicating the ability to reflect the link of age to exercise levels. Furthermore, the BMI values within the sample are concentrated within a specific range, exhibiting minimal differences, which hinders the observation of any potential relationship with exercise levels.

This study, while not directly comparing the sleep quality of students across various colleges and universities, highlights a significant concern regarding sleep disorders among college students. The data presented herein reveal discrepancies in the relationship between exercise and sleep among college students when compared to findings from other studies (31). These variations may be linked to differences in geographic location, institutional environment, and the professional characteristics of the participants. For instance, a substantial portion of the sample comprised medical students, whose demanding curricula may be associated with their sleep patterns. Additionally, the availability and quality of sports facilities at the institution may be correlated with students’ levels of physical activity. Consequently, it is imperative that health education strategies are tailored to reflect the specific conditions of each institution and are adapted to local circumstances. The study by Zhihao et al. (32) revealed the mediating roles of self-efficacy and self-control in the relationship between physical activity and internet addiction among college students, whereas the present study focuses on the role of sleep quality and stress in the pathway through which physical activity exerts its influence. Although both studies indicate that physical activity promotes positive outcomes—such as improved sleep, reduced stress, or decreased internet addiction—their underlying mechanisms differ. The former emphasizes the mediating pathway of self-regulatory capacity, while the present study highlights the mediating effect of physiological-psychological states (sleep and stress), suggesting that physical activity may exert beneficial effects on diverse health outcomes through distinct psychological pathways.

The research by Du et al. (33) further identified the mediating role of self-esteem and the moderating effect of gender, noting that male students generally engaged in higher levels of physical activity than females, which is consistent with our finding that males participated more frequently in moderate- to high-intensity sports. This commonality suggests that gender differences in physical activity participation are stable across samples. Moreover, both studies support the notion that physical activity can indirectly influence target outcomes (such as internet addiction or sleep quality) through psychological variables, further underscoring the key role of psychological mechanisms in the relationship between physical activity and health behaviors.

Regarding the association between physical activity and sleep quality, results from multiple studies (33–36) are consistent. For instance, one study (34) indicated that meeting the 24-h movement guidelines significantly reduced the risk of poor sleep quality (AOR = 0.33), which aligns with the negative correlation between sports participation and sleep quality (r = −0.222) observed in our study, suggesting that the beneficial effect of physical activity on sleep is generalizable across cultural contexts. Another study (37) focusing on the specific dimension of “restful sleep” found it to be significantly associated with physical activity, thereby complementing our overall assessment of sleep quality at a more granular level. However, as that study sampled primarily non-Hispanic white students from the northeastern United States, differences in demographic composition may affect direct comparisons of the strength of association.

Concerning the mediating role of stress, one study (38) revealed that stress and subjective well-being acted as chain mediators between physical activity and sleep quality, while our study also showed a significant negative correlation between stress and physical activity (r = −0.250), as well as a negative influence of stress on sleep quality. Both sets of findings support the view that “stress is an important mediator in the relationship between physical activity and sleep quality,” highlighting the importance of stress management in promoting healthy behaviors among college students.

To enhance the physical and mental well-being of college students, higher education institutions should implement a multifaceted approach. Firstly, it is essential to optimize curricula and judiciously allocate the number of courses for each academic year to prevent excessive academic demands from being linked to adverse effects on students’ physical activity and sleep patterns. Secondly, institutions should prioritize health education by disseminating information regarding the significance of exercise and sleep for overall health, thereby increasing students’ health awareness. Additionally, a variety of sports activities should be organized to encourage active student participation, fostering a supportive environment for physical engagement. To address sleep-related issues, institutions may conduct lectures focused on sleep hygiene to assist students in developing healthy sleep habits.

Although the correlation coefficients between physical activity and sleep quality/stress levels found in this study are relatively low (ranging from −0.22 to −0.25), falling within the weak correlation range, this does not diminish their practical significance. In research involving complex human behaviors and psychological states, strong correlations between variables are uncommon due to the influence of multiple biological, psychological, social, and environmental factors. Even small effect sizes can hold important public health implications at the population level.

### 4.2 Limitations

This study employed a cross-sectional research methodology, which identified correlations between variables but did not establish causality. For instance, while the findings indicated an association between exercise levels and both sleep quality and stress, it remains unclear whether exercise is linked to sleep and stress, whether sleep and stress are associated with exercise, or whether other complex causal relationships exist. To accurately ascertain causal relationships, future research should adopt cohort studies or experimental epidemiological designs that monitor changes in various factors over time. Furthermore, this study addressed only a subset of lifestyle factors and did not consider other significant elements, such as psychological, socio economic situation，physiological, and nutritional factors. The sleep and exercise behaviors of college students are associated with a multitude of factors, including psychological aspects like personality traits and cognitive styles, physiological elements such as hormone levels and genetic predispositions, and nutritional considerations including dietary habits and nutrient intake. However, the current study did not investigate these dimensions in sufficient depth, resulting in findings that are not comprehensive enough to fully elucidate the mechanisms linked to college students’ health behaviors. The research sample was solely drawn from a medical college in China and mainly consisted of medical students. The academic characteristics and lifestyle rhythms of medical students differ significantly from those of non - medical students. Meanwhile, the economic level and cultural background of the region where the college is located may also influence health behaviors. As a result, it is difficult to generalize the research findings to students in other majors and other types of institutions (such as comprehensive universities and vocational colleges). It is important to acknowledge that this study relies on self-reported data collected via questionnaires including physical activity, stress, and sleep quality assessments. Self-reported data may introduce reporting bias and recall bias, which could potentially affect the accuracy of the study results. And this study relies on a convenience sample from a single university, which may limit the generalizability of the findings to college students from other institutions or regions.

## 5 Conclusion

In conclusion, the sleep and exercise patterns of college students are associated with a multitude of factors. It is imperative that colleges, universities, and educational departments acknowledge this issue and implement effective strategies to enhance the current situation. Such measures will support college students in achieving optimal physical and mental health development. Furthermore, the observed gender disparity in participation rates for moderate-to-vigorous physical activity—with male students engaging at significantly higher levels—may also reflect deeply embedded sociocultural expectations. In many contexts, sports and vigorous exercise are still perceived as masculine domains, while females may face implicit or explicit social pressures that discourage them from pursuing such activities. These pressures can include gender stereotypes regarding physical ability, concerns about body image, or a lack of female role models in sports. To address these barriers, future research should explicitly examine how gendered social norms influence motivation, self-perception, and access to sports among female students. Additionally, interventions aimed at promoting physical activity should be gender-sensitive, for instance, by offering diverse forms of exercise that appeal to different preferences, creating supportive female-only sports environments, and integrating body-positive messaging into health education. By doing so, institutions can help mitigate the impact of restrictive social expectations and foster more equitable participation in physical activity.

## Data Availability

The original contributions presented in the study are included in the article/Supplementary material, further inquiries can be directed to the corresponding author.
